# Frequency of Cholelithiasis in Patients With Beta-Thalassemia Intermedia With and Without Hydroxyurea

**DOI:** 10.5812/ircmj.18712

**Published:** 2014-07-05

**Authors:** Maryam Khavari, Azin Hamidi, Sezaneh Haghpanah, Mohammad Hadi Bagheri, Marzieh Bardestani, Razieh Hantooshzadeh, Mehran Karimi

**Affiliations:** 1 Department of Biology, Faculty of Sciences, Zabol University of Medical Sciences, Zabol, IR Iran; 2 Hematology Research Center, Shiraz University of Medical Sciences, Shiraz, IR Iran; 3 Medical Imaging Research Center, Shiraz University of Medical Sciences, Shiraz, IR Iran; 4 Department of Library and Information Sciences, College of Humanities, Khouzestan Science and Research Branch, Islamic Azad University, Ahvaz, IR Iran; 5 Ministry of Health and Medical Education, Tehran, IR Iran

**Keywords:** Beta Thalassemia Intermedia, Cholelithiasis, Hydroxyurea

## Abstract

**Background:**

Recent studies regarding the effect of hydroxyurea (HU) in thalassemia have revealed favorable effects on the reduction of ineffective erythropoiesis.

**Objectives:**

The aim of the current study was to evaluate whether or not HU can have an effect on the gallstone formation rate in patients with beta-thalassemia intermedia (BTI).

**Patients and Methods:**

In this case control cross-sectional study, from a total of 250 transfusion-independent BTI patients, 51 patients who were taking HU, participated in the study. Patients were registered in the Thalassemia clinic of Shiraz University of Medical Sciences, Shiraz, which is a referral center located in southern Iran, during 2011-2012. Mean dose of HU consumption in the case group was 10 ± 2.5 mg/kg/day (range of 8-15 mg/kg/day), with a mean duration of consumption of 7.5 ± 3.8 years (range 1-14 years). In addition, 41 age- and sex-matched BTI patients who did not use HU were randomly selected as a control group. All patients underwent abdominal ultrasound by a radiologist for evaluation of gallstones.

**Results:**

Mean age of the participants was 21.4 ± 6.5 years (10-40 years). There was no statistically significant difference regarding the frequency of cholelithiasis between the two groups of patients (P = 0.822). Our study showed significantly lower hemoglobin levels and a higher percentage of nucleated red blood cells in the HU group compared with the control group (P = 0.001 and P = 0.005, respectively).

**Conclusions:**

It seems that taking HU for long periods can reduce hemolysis and bone marrow suppression, and that decreases the rate of cholelithiasis. We believe that if these patients had not been treated with HU, we would probably have observed a significantly higher frequency of cholelithiasis, due to more hemolysis compared with patients not taking HU. Further studies with larger sample sizes are suggested.

## 1. Background

Thalassemia is a monogenic hematologic disorder with a high prevalence in Iran, mostly in the southern part of the country (1). This disorder is categorized into α and β types, based on the abnormal globin chain and each are divided into major, intermedia and minor subtypes regarding the severity of chain abnormality (2). Beta-thalassemia intermedia (BTI) is defined by multiple mutations and results in thalassemia symptoms at a moderate intensity, less than beta thalassemia major (BTM) (3, 4). These patients may not develop any symptoms until adulthood as they can maintain their hemoglobin levels between 7-10 g/dL, and as a result there would also be less need for blood transfusions (1-5). Thalassemia patients may face multiple complications with their disease, such as; hepatomegaly, splenomegaly, pulmonary hypertension and anemia due to hemolysis and ineffective erythropoiesis. Gall stones are described as one of the most prevalent complications of thalassemia. Gallstone formation is mostly due to iron deposition, followed by hemolysis and ineffective erythropoiesis, which results in gastrointestinal symptoms in patients and frequently results in a cholecystectomy (5-8). Hydroxyurea (HU) is an S phase specific and non-DNA hypomethylating agent, which is routinely used in myeloproliferative disorders and sickle cell anemia, and it is believed to induce hemoglobin F and globin chain synthesis (9, 10), thus it can prevent ineffective erythropoiesis (8-13). Recent studies on the effects of HU in thalassemia patients have revealed favorable effects on ineffective erythropoiesis and this probably results in decreased gallstone formation in these patients (3, 6, 7).

## 2. Objectives

The aim of the current study was to investigate whether or not HU has an effect on gallstone formation rates in patients with thalassemia intermedia.

## 3. Patients and Methods

In this case control cross-sectional study, out of a total of 250 transfusion-independent BTI patients, 51 patients who were taking HU, participated in this study. These patients were registered in the Thalassemia clinic of Shiraz University of Medical Sciences, Shiraz, which is a referral center in southern Iran, during 2011 and 2012. Mean dose of HU consumption in the case group was 10 ± 2.5 mg/kg/day (range 8-15 mg/kg/day). All of these patients maintained good compliance with HU and the mean duration of consumption was 7.5 ± 3.8 years (range 1-14 years). Patients, who had cholelithiasis before taking HU, were excluded from the study. Furthermore, we randomly selected 50 age- and sex-matched BTI patients, who did not use HU, as a control group. Nine patients in this group did not complete the laboratory examination and they were excluded from the study. Finally, 41 patients participated as controls. Informed written consent was taken from all participants. This study was approved by the Ethics Committee of Shiraz University of Medical Sciences (code number = 2411, date: April 2011). Diagnosis of BTI was based on clinical history, complete blood count and hemoglobin electrophoresis. An ad-hoc questionnaire was used for gathering all of the demographic and laboratory data and clinical characteristics of the patients, including; age, sex, duration of HU use, size of liver and spleen, hemoglobin, nucleated red blood cells, ferritin, alanine transaminase (ALT), aspartate transaminase (AST), direct and total bilirubin. All laboratory data was taken from the last data of their medical records. Physical examinations included the size of the liver and spleen. The liver and spleen sizes of the patients were categorized as; less than 6 cm, equal to, or more than 6 cm below the costal margin. All patients underwent abdominal ultrasound by a radiologist for evaluation of gallstones by an Accuvix 10 ultrasound device (Medison Co Ltd. Seoul, South Korea).

### 3.1. Statistical Analysis

Data analysis was done by Statistical Package for the Social Sciences (SPSS, Chicago, Illinois, USA) software version 17. Normality of data was checked by a Shapiro-Wilk test. Student’s t-test and Mann-Whitney-U test were carried out for the comparison of quantitative variables. Qualitative variables between the two groups were compared by Chi-square test. P-value less than 0.05 was considered as statistically significant.

### 3.2. Sample Size

Based on a pilot study, about 32% and 12% of BTI patients develop gallstones, with and without taking HU, respectively. By using α = 0.05 and power = 70%, 50 patients were calculated in each group for our study. Thus, in this study all patients with HU therapy (51 patients) were included and we randomly selected 50 age-and sex-matched patients who were not taking HU for the control group. Nine patients in this group did not complete a laboratory examination and were excluded from the study (41 patients).

## 4. Results

Mean age of the participants was 21.4 ± 6.5 years (10-40 years), including 46 females (50%) and 46 males (50%). Comparison of demographic data and clinical characteristics of the patients with and without using HU are summarized in Table 1.

**Table 1.** Comparison of Demographic and Laboratory Data of Beta Thalassemia Intermedia Patients With and Without Hydroxyurea^a,b^

Variables	Patients With Hydroxyurea Therapy, n = 51	Patients Without Hydroxyurea Therapy, n = 41	P value
**Age, y**			0.498
Mean ± SD	21.9 ± 0.7	20.9 ± 0.8	
Median	21.5	21	
IQR	8	7	
**Gender**			0.999
Male	26	20	
Female	25	21	
**Hb, g/dL**			0.001^c^
Mean ± SD	8.7 ± 1.2	9.6 ± 1.3	
Median	8.6	9.7	
IQR	1.5	1.9	
**NRBC**			0.021^c^
Mean ± SD	4.3 ± 3.5	2.7 ± 1.7	
Median	3	2	
IQR	4.3	1.5	
**Ferritin, μg/L**			0.577
Mean ± SD	583 ± 433	782 ± 966	
Median	423	460	
IQR	651	360	
**AST, u/L**			0.183
Mean ± SD	33.8 ± 24.3	28.2 ± 12.4	
Median	30	25	
IQR	17	13	
**ALT, u/L**			0.308
Mean ± SD	22.9 ± 32.6	18.8 ± 17.9	
Median	14	13	
IQR	12	10	
**Total bilirubin, mg/dL**			0.729
Mean ± SD	3.2 ± 1.3	3.5 ± 2.5	
Median	3	2.8	
IQR	1.7	2.2	
**Direct bilirubin, mg/dL**			0.536
Mean ± SD	0.57 ± 0.34	0.51 ± 0.21	
Median	0.5	0.5	
IQR	0.3	0.3	
**Liver size, ≥ 6 cm**	39 (76.5)	23 (56)	0.053
**Spleen size** ^**d**^ **, ≥ 6 cm**	19 (37.3)	24(58.5)	0.764
**Splenectomy, yes**	25 (49)	10 (24.4)	0.018^c^
**Cholelithiasis**	17 (33.3)	12 (29.3)	0.822

^a^ Abbreviations: Hb, hemoglobin; NRBC, nucleated red blood cells; IQR, Interquartile Range; SGOT, serum glutamic oxaloacetic transaminase; SGPT, serum glutamic pyruvic transaminase.

^b^ Data are presented as Mean ± SD or No. (%).

^c^ Statistically significant.

^d^ Data of spleen size has been related to 57 non-splenectomized patients.

The rate of cholelithiasis was not significantly different between the two groups (33.3% in the HU group vs. 29.3% in the control group, P = 0.677). Significantly lower hemoglobin levels and higher percentages of nucleated RBCs (NRBC) were found in the HU group compared with the control group (P = 0.001 and P = 0.021, respectively). But none of these two parameters showed a significant correlation with cholelithiasis (P = 0.170 for hemoglobin and P = 0.225 for NRBC). A statistically significant higher frequency of splenectomy was observed in the HU group compared with the controls (49% vs. 24.4%, P = 0.018). Splenectomy was also significantly associated with cholelithiasis (55.2% in patients with cholelithiasis vs. 30.2% in patients without cholelithiasis, P = 0.036); however, after stratification with regard to splenectomy, there was no significant association found between HU taking and cholelithiasis (P = 0.723 in splenectomized patients and P = 0.753 in non-splenectomized patients). There were no statistically significant differences with regard to other data, including; demographic and laboratory data, and liver and spleen size, or between the two groups of patients with and without taking HU (Table 1). Patients with cholelithiasis were significantly older than those without cholelithiasis (24.5 ± 6.5 vs. 20 ± 6.1, P = 0.003). There was not any significant relationship between cholelithiasis and sex (P = 0.654), or ferritin level (P = 0.850).

## 5. Discussion

We evaluated the frequency of cholelithiasis in patients with BTI who were taking HU and those who were not. According to previous results, HU with a mean dose of 10 ± 2.5 mg/kg/day can have a positive effect on increasing hemoglobin levels and decreasing the percentage of NRBC (14, 15). However, in comparison with patients who were not taking HU, as observed in this study, patients with HU could have lower Hb levels due to a more severe clinical condition at the start of treatment. Koren et al. (16) and Italia et al. (15) found that HU consumption was significantly associated with a decreased need for blood transfusion in patients with BTI. Based on our results, the rate of gallstones was not significantly different between patients who were taking HU therapy and those who were not. Laboratory data such as ferritin level, AST, ALT and bilirubin (direct and total) were not significantly different between the two groups of patients. The higher rate of splenectomy in the HU group might be due to the lower clinical condition of these patients, such as hypersplenism, which led to a splenectomy. HU was also determined to be safe and without significant adverse effects during a 10 year duration of taking HU, in a study that was published in 2010 (17). The effect of HU on the prevalence of cholelithiasis was evaluated in the north of Iran. A total of 72 patients with beta thalassemia major and intermedia, with a mean age of 22.4 ± 7.5 years, included 36 patients who were using HU with a mean dose of 14.9 ± 5.9 mg/kg/day and a mean duration of 67.9 ± 25.5 months. Abdominal ultrasound revealed that 48% of the patients who did not use HU and 19.4% of patients who were using HU had cholelithiasis, which was statistically significant (6). In our study, the HU group had more severe forms of the disease (more hemolysis and ineffective erythropoiesis, more NRBC, and lower Hb levels), subsequently we expected a higher rate of cholelithiasis in this group. However, we did not observe a significantly higher rate of gallstones in these patients. We hypothesized that HU can have a positive effect on decreasing hemolysis and cholelithiasis in this group. In order to achieve a higher effect on decreasing the rate of cholelithiasis, a higher dose of HU may be needed as prescribed in another study (6). In addition, the genetic background can have some effects in this regard. Further evaluation regarding the effects of HU on cholelithiasis in thalassemia patients is required. Our study was limited due to the more severe clinical condition in the patients on HU therapy compared with the controls; however, we evaluated confounding variables between the two groups and there was no statistically significant difference with regard to hemoglobin level, percentage of NRBC, and other laboratory data. Another limitation of our study was the small sample size in each group of our study (with and without taking HU). It seems that to improve an evaluation of the relationship between HU consumption and gallstone, further larger studies are needed. Our study was the first study to evaluate the relationship of HU consumption and cholelithiasis in patients with BTI. In conclusion, because patient candidates for HU therapy had a worse clinical condition for longer periods regarding hemolysis, we expected a significantly higher rate of cholelithiasis in this group, but we observed an insignificantly difference of gallstone frequency in this group compared with patients without taking HU. It seems that taking HU at a dosage of 10 ± 2.5 mg/kg/day for a long duration of about 7.5 ± 3.8 years may reduce hemolysis and bone marrow suppression which results in lower rates of cholelithiasis.

## References

[1] Abolghasemi H, Amid A, Zeinali S, Radfar MH, Eshghi P, Rahiminejad MS (2007). Thalassemia in Iran: epidemiology, prevention, and management.. J Pediatr Hematol Oncol..

[2] Kliegman R, Stanton B, Behrman R, Schor N, St.geme J. Nelson textbook of pediatrics..

[3] Karimi M, Haghpanah S, Farhadi A, Yavarian M (2012). Genotype-phenotype relationship of patients with beta-thalassemia taking hydroxyurea: a 13-year experience in Iran.. Int J Hematol..

[4] Taher A, Isma'eel H, Cappellini MD (2006). Thalassemia intermedia: revisited.. Blood Cells Mol Dis..

[5] Mishra AK, Tiwari A (2013). Iron overload in Beta thalassaemia major and intermedia patients.. Maedica (Buchar)..

[6] Karami H, Vahidshahi K, Kosarian M, Karami H, Jamali M (2007). Relationship between Use of Hydroxyurea and Cholelithiasis in Patients with Major and Intermediate β-thalassemia.. J Mazandaran Univ Med Sci..

[7] Kosaryan M, Karami H, Zafari M, Yaghobi N (2014). Report on patients with non transfusion-dependent beta-thalassemia major being treated with hydroxyurea attending the Thalassemia Research Center, Sari, Mazandaran Province, Islamic Republic of Iran in 2013.. Hemoglobin..

[8] Musallam KM, Taher AT, Rachmilewitz EA (2012). beta-thalassemia intermedia: a clinical perspective.. Cold Spring Harb Perspect Med..

[9] Taher A, Sheikh-Taha M (2006). Hydroxyurea use in Lebanese patients with beta-thalassemia intermedia.. J Pediatr Hematol Oncol..

[10] Borgna-Pignatti C (2007). Modern treatment of thalassaemia intermedia.. Br J Haematol..

[11] Kattamis A (2007). Treatment of thalassemia with hydroxyurea: an indispensable alternative therapy.. J Pediatr Hematol Oncol..

[12] Patel S, Purohit P, Mashon RS, Dehury S, Meher S, Sahoo S (2014). The effect of hydroxyurea on compound heterozygotes for sickle cell-hemoglobin D-Punjab-A single centre experience in eastern India.. Pediatr Blood Cancer..

[13] Silva DG, Belini Junior E, Carrocini GC, Torres Lde S, Ricci Junior O, Lobo CL (2013). Genetic and biochemical markers of hydroxyurea therapeutic response in sickle cell anemia.. BMC Med Genet..

[14] Ehsani MA, Hedayati-Asl AA, Bagheri A, Zeinali S, Rashidi A (2009). Hydroxyurea-induced hematological response in transfusion-independent beta-thalassemia intermedia: case series and review of literature.. Pediatr Hematol Oncol..

[15] Italia KY, Jijina FJ, Merchant R, Panjwani S, Nadkarni AH, Sawant PM (2009). Response to hydroxyurea in beta thalassemia major and intermedia: experience in western India.. Clin Chim Acta..

[16] Koren A, Levin C, Dgany O, Kransnov T, Elhasid R, Zalman L (2008). Response to hydroxyurea therapy in beta-thalassemia.. Am J Hematol..

[17] Karimi M, Cohan N, Mousavizadeh K, Falahi MJ, Haghpanah S (2010). Adverse effects of hydroxyurea in beta-thalassemia intermedia patients: 10 years' experience.. Pediatr Hematol Oncol..

